# Metabolic instability vs fibre recruitment contribution to the ${\dot{V}O_2}$ slow component in different exercise intensity domains

**DOI:** 10.1007/s00424-021-02573-8

**Published:** 2021-05-19

**Authors:** Alessandro L Colosio, Kevin Caen, Jan G. Bourgois, Jan Boone, Silvia Pogliaghi

**Affiliations:** 1 Department of Neurosciences, Biomedicine and Movement Sciences, University of Verona, Via Casorati 43, 37131 Verona, Italy; 2 Department of Movement and Sports Sciences, Ghent University, Watersportlaan 2, Ghent, Belgium

**Keywords:** Oxidative metabolism, ${\dot{V}O_2}$ kinetics, Oxygen consumption, Excess ${\dot{V}{O}_2}$, Loss of efficiency

## Abstract

This study focused on the steady-state phase of exercise to evaluate the relative contribution of metabolic instability (measured with NIRS and haematochemical markers) and muscle activation (measured with EMG) to the oxygen consumption (${\dot{V}O_2}$) slow component (${\dot{V}O_2}{_s}{_c}$) in different intensity domains. We hypothesized that (i) after the transient phase, ${\dot{V}O_2}$, metabolic instability and muscle activation tend to increase differently over time depending on the relative exercise intensity and (ii) the increase in ${\dot{V}O_2}{_s}{_c}$ is explained by a combination of metabolic instability and muscle activation. Eight active men performed a constant work rate trial of 9 min in the moderate, heavy and severe intensity domains. ${\dot{V}O_2}$, root mean square by EMG (RMS), deoxyhaemoglobin by NIRS ([HHb]) and haematic markers of metabolic stability (i.e. [La^−^], pH, HCO_3_^−^) were measured. The physiological responses in different intensity domains were compared by two-way RM-ANOVA. The relationships between the increases of [HHb] and RMS with ${\dot{V}O_2}$ after the third min were compared by simple and multiple linear regressions. We found domain-dependent dynamics over time of ${\dot{V}O_2}$, [HHb], RMS and the haematic markers of metabolic instability. After the transient phase, the rises in [HHb] and RMS showed medium–high correlations with the rise in ${\dot{V}O_2}$ ([HHb] *r* = 0.68, *p* < 0.001; RMS *r* = 0.59, *p* = 0.002). Moreover, the multiple linear regression showed that both metabolic instability and muscle activation concurred to the ${\dot{V}O_2}{_s}{_c}$ (*r* = 0.75, [HHb] *p* = 0.005, RMS *p* = 0.042) with metabolic instability possibly having about threefold the relative weight compared to recruitment. Seventy-five percent of the dynamics of the ${\dot{V}O_2}{_s}{_c}$ was explained by [HHb] and RMS.

## Introduction

During constant work rate exercise (CWR), oxygen consumption (${\dot{V}O_2}$) response is linearly related to the workload with a ratio of around ~ 10 ml*min^−1^*W^–1^ [23]. However, as exercise intensity increases, and particularly at intensities above the gas exchange threshold (GET), this relationship is lost and a further, theoretically unexpected, increase in ${\dot{V}O_2}$ is detectable. This increase in ${\dot{V}O_2}$, defined as the “slow component” of ${\dot{V}O_2}$ (${\dot{V}O_2}{_s}{_c}$), as it manifests ~ 3 min after exercise onset, is usually interpreted as an increased O_2_ cost of locomotion [24]. Typically, when exercise is performed in the heavy intensity domain, between GET and the respiratory compensation point (RCP, or the critical power), ${\dot{V}O_2}{_s}{_c}$ tends to a steady state. On the contrary, when effort rises above RCP (i.e. severe exercise domain), a steady state is not achievable and ${\dot{V}O_2}$ continues to increase over time to finally reach the maximal oxygen consumption (${\dot{V}O_2}{_m}{_a}{_x}$) [24].

Of the ${\dot{V}O_2}{_s}{_c}$ recorded at the mouth, around 85% originates from the contracting muscles, while the remaining 15% is caused by the increased cost of ventilation [31]. Considering the muscular component of the ${\dot{V}O_2}{_s}{_c}$, it was proposed that either the recruitment of less efficient type II motor units necessary to maintain a specific power output [8, 9, 24] or metabolic instability occurring within the working fibres [36, 39] could represent the main physiological underpinnings. In both scenarios, muscle contractions become less efficient, therefore requiring a higher energetic demand in order to maintain the same external power output. Moreover, Grassi et al. (2015) have proposed that as exercise is protracted, these two phenomena may mutually influence each other in a vicious circle in which the changes in homeostasis of the working muscle lead to loss of efficiency, which in turn may cause the recruitment of larger and less efficient motor units, as such further affecting metabolic stability [17].

Adding complexity, recent studies [10, 30] questioned the very existence of a ${\dot{V}O_2}{_s}{_c}$ by applying a method that accounts for the ${\dot{V}O_2}$ cost of ventilation and the ${\dot{V}O_2}$ equivalent of lactate accumulation. Analysing the response between different intensity domains, the authors [10] suggested that in the heavy domain, the observed increase in ${\dot{V}O_2}$ over time could be mainly the result of a delayed adjustment of ${\dot{V}O_2}$, while only in severe domain a “true” loss of efficiency manifests. It was speculated that the recruitment of intrinsically less efficient and fatigable type II fibres could explain the loss of efficiency reported in the severe intensity domain [8, 17, 24].

To date, the ${\dot{V}O_2}{_s}{_c}$ in heavy and severe was treated as equivalent and, as a consequence, the literature investigating the origins of the slow component often tested only one exercise intensity (heavy or severe) and either muscular metabolic instability or fibre recruitment (mostly due to complexity or economic reasons). However, given that the ${\dot{V}O_2}{_s}{_c}$ may not entail a loss of efficiency of locomotion in heavy but only in severe [10], a study aimed at testing its muscular contributors at different intensities would add valuable information to the ongoing debate.

In this context, near-infrared spectroscopy (NIRS) provides a non-invasive index of oxygen extraction that reflects the imbalance between delivery and utilization within the working muscle [18, 19] and in turn may be associated with metabolic instability [17]. Furthermore, electromyography (EMG) allows an indirect estimate of motor units activation during muscle contraction. Interestingly, while the use of these techniques has increased exponentially during the past two decades, no study has applied them simultaneously to gain insight into the origins of the ${\dot{V}O_2}{_s}{_c}$ across exercise domains.

Therefore, this investigation was designed to focus on the steady-state phase of exercise by evaluating the relative contribution of metabolic instability (measured with NIRS and haematochemical markers of metabolic balance/homeostasis) and muscle activation to the ${\dot{V}O_2}{_s}{_c}$. We tested the following hypotheses: (i) after the initial 3 min, ${\dot{V}O_2}$, metabolic instability and muscle activation display a different tendency to increase over time depending on the relative exercise intensity (i.e. no changes occur in the moderate domain and increasing dynamics are observed in the heavy and severe intensity domains) and (ii) the increase in ${\dot{V}O_2}{_s}{_c}$ is explained by a combination of metabolic instability and muscle activation.

## Methods

### Ethical approval

The study was conducted according to the Declaration of Helsinki and all procedures were approved by the University of Verona Ethics Committee for Research on Human Subjects. Procedures and risks were explained to each subject, and all participants volunteered and gave informed written consent to participate before the start of the study.

### Participants

Eight active men were recruited in the study (age 25 ± 2 years, body mass 74 ± 10 kg, height 181 ± 5 cm, ${\dot{V}O_2}{_p}{_e}{_a}{_k}$: 3643 ± 457 ml*min^−1^, 49 ± 3 ml*min^−1^*kg). Inclusion criteria were male sex and age between 20 and 35 years; exclusion criteria were smoking and any condition that could influence the physiological responses during testing. All participants were instructed to avoid caffeine consumption and physical activity respectively for at least 8 h and 24 h before each testing session. Moreover, to minimize variability of glycogen stores and glucose oxidation, participants followed a standard food intake prescription before all the testing sessions as previously described [10, 16] (i.e. 2 g of low glycemic index carbohydrates per kilogram of body weight, 2 h before testing; 0.5 L of water in the 90 min before testing; restriction from caffeine during the 8 h before testing).

### Experimental protocol

${\dot{V}O_2}$, EMG and NIRS data for this investigation were collected during the 9-min trials of a previous investigation [10] in which participants performed 3 tests (respectively of 3, 6 and 9 min) in every exercise intensity domain within a maximum of 4 weeks. Briefly, all subjects completed (i) a preliminary maximal ramp incremental exercise test to exhaustion for the determination of GET, RCP and the peak of oxygen uptake (${\dot{V}O_2}{_p}{_e}{_a}{_k}$); (ii) three CWR trials in the moderate exercise intensity domain; (iii) three CWR in the heavy exercise intensity domain and (iv) three CWR trials in the severe exercise intensity domain [13]. Moreover, the haematic response to exercise (i.e. blood lactate ([La^−^]) accumulation, pH, bicarbonate (HCO_3_^−^)) was characterized at baseline and every 3 min, by taking blood samples at the 1st, 3rd, 5th and 7th min after exercise stop of the 3-, 6- and 9-min CWR respectively. Tests were executed in randomized order with the only exception of the longest CWR in the “severe” exercise domain that was completed as first to assure that subjects were able to sustain the power output for the required time. All exercise tests were conducted on an electromagnetically braked cycle ergometer (Sport Excalibur, Lode, Groningen, the Netherlands), at a similar time of the day in an environmentally controlled laboratory (18 °C, 55–65% relative humidity).

### Ramp incremental test

The ramp incremental test consisted of a 3-min baseline cycling at 50 W, followed by a 30-W*min^−1^ increase in power output until volitional exhaustion. Participants were asked to pick a self-selected cadence in the range of 70–90 rpm and to maintain it throughout all subsequent tests. Failure to maintain the indicated cadence within 5 rpm (for longer than 5 s) during testing despite strong verbal encouragement was considered as the criterion for exhaustion. Breath-by-breath pulmonary gas exchange and ventilation were continuously measured using a metabolic cart (Jaeger Oxycon Pro, Viasys Healtcare GmbH, Höchberg, Germany) as previously described [12]. Heart rate (HR) was monitored continuously (H7 Sensor, Polar, Kempele, Finland).

### Constant work rate trials

After the preliminary ramp incremental test, subjects completed 3 CWR trials within each exercise intensity domain (i.e. moderate at 80% of GET, heavy at 50%Δ between GET and RCP, and severe at 60%Δ between GET and ${\dot{V}O_2}{_p}{_e}{_a}{_k}$) in a randomized order. Each CWR was preceded by a 3-min warm-up at 20 W. Throughout the test, subjects kept the same, constant rpm and bike position as selected during the ramp incremental test. ${\dot{V}O_2}$ and HR data were measured with the same method described for the ramp incremental test. Capillary blood samples (65 μl) were drawn from the fingertip in the last 30 s of warm-up and at the 1st, 3rd, 5th and 7th min after each test and were immediately analysed using a benchtop blood analyser (Radiometer ABL90 FLEX, Radiometer Medical ApS, Brønshøj, Denmark) to measure [La^−^], pH and HCO_3_^−^. The highest value of [La^−^] was considered as the peak of blood lactate concentration and used for further analysis. The blood sample with the highest [La^−^] was also used to define pH and HCO_3_^−^ at a given time point.

Deoxygenation of the left *vastus lateralis* was evaluated in microcirculation using a quantitative near-infrared spectroscopy system (OxiplexTS, ISS, Champaign, USA) that provided continuous measurement (sampling frequency 1 Hz) of the absolute concentrations (μM) of deoxyhaemoglobin ([HHb]). After shaving, cleaning and drying of the skin area, the NIRS probe was positioned longitudinally on the belly of the vastus lateralis muscle ~ 15 cm above the patella, attached to the skin with a bi-adhesive tape and secured with elastic bandages around the thigh. The device was calibrated before each test after a warm-up of at least 30 min as per manufacturer recommendations.

Surface EMG of the right *vastus lateralis* muscle was continuously recorded by means of a wireless system (1500 Hz; ZeroWire, Noraxon, Scottsdale, AZ, USA). A pair of surface Ag/AgCl electrodes (Blue sensor, Ambu®, Ballerup, Denmark) was attached to the skin with a 2-cm inter-electrode distance. The electrodes were placed longitudinally with respect to the underlying muscle fibres arrangement, according to the recommendations by Surface EMG for non-invasive assessment of muscles [20]. Before electrode application, the skin was shaved, scratched with sand-paper and cleaned with alcohol in order to minimize impedance. Semi-permanent ink marks allowed consistent re-positioning of the electrodes between sessions. The EMG transmitter connected to the electrodes was well secured with adhesive tape to avoid movement-induced artefacts.

### Data analysis

#### Ramp incremental test

For the gas exchange variables, aberrant data points that lay 3 SD from the local mean were removed and were linearly interpolated on a 1-s basis and then averaged every 5 s. ${\dot{V}O_2}{_p}{_e}{_a}{_k}$ was determined as the highest ${\dot{V}O_2}$ obtained over a 10-s interval [15]. GET and RCP were determined by three blinded experts as detailed elsewhere [15]. Briefly, GET was determined by visual inspection as the ${\dot{V}O_2}$ at which CO_2_ output began to increase out of proportion in relation to ${\dot{V}O_2}$, with a systematic rise in the ventilation $(VE)-to-{\dot{V}O_2}$ relation and end-tidal PO_2_, whereas the ventilatory equivalent of VCO_2_ (VE/VCO_2_) and end-tidal PCO_2_ is stable [2]. RCP was determined as the point where end-tidal PCO_2_ began to fall after a period of isocapnic buffering [38]. This point was confirmed by examining VE/VCO_2_ plotted against ${\dot{V}O_2}$ and by identifying the second breakpoint in the $VE-to-{\dot{V}O_2}$ relation. Finally, we determined the workloads equivalent to the specific moderate (80% of GET), heavy (50% Δ between GET and RCP) and severe (60%Δ between GET and ${\dot{V}O_2}{_p}{_e}{_a}{_k}$) ${\dot{V}O_2}$ targets. To this aim, the ${\dot{V}O_2}/W$ relationship identified in the incremental test was left shifted to account for the mean response time [15].

#### Constant work rate trials

${\dot{V}O_2}$ during CWR was sampled breath-by-breath, interpolated using the same procedure described for the ramp incremental test and time aligned with the onset of exercise. To isolate ${\dot{V}O_2}$ contributing to locomotion (${\dot{V}O_2}{_m}$), the ${\dot{V}O_2}$ requested by ventilation was subtracted from the ${\dot{V}O_2}$ measured at the mouth level as broadly described elsewhere [10] In brief, the work of breathing was calculated based on VE using the equation by Coast et al.:

$$\rm{Work\, of\, breathing}= -0.430+0.050*VE+0.00161\,V{E}^{2}$$

Then, the work of breathing was used to calculate the amount of ${\dot{V}O_2}$ requested by ventilatory muscles [7]:

$$\dot{\rm{V}}\rm{O}_{2} \, \rm {requested \,by\,ventilation}=34.9+7.45*\rm work\,of\,breathing$$

The NIRS-derived [HHb] response of the three trials within each domain was time aligned and averaged with the onset of exercise transition and treated by subtracting the steady-state value measured during the last 2 min of warm-up.

Raw EMG signal was rectified and smoothed using a fourth-order band-pass Butterworth digital filter with a frequency range set between 20 and 500 Hz. Root mean square (RMS) was calculated every second from the raw signal and was used as an index of the total muscle excitation for *vastus lateralis* [37]. Thereafter, the RMS recorded during the last 2 min of 20 W baseline for each test was used to normalize the CWR and expressed as multiples of baseline [29].

Then, the slopes of increase of each signal were calculated (i.e. ${\dot{V}O_2}{_m}$, [HHb], RMS, [La^−^], pH and HCO_3_^−^) in the time window between the 180 and 540 s after exercise starts. Based on the prediction of the time to a steady state of this sample of subjects (calculated as the time delay (25 ± 2 s) + 4 * tau (30 ± 3 s)), 180 s was chosen as the minimum time to achieve a steady state in all the intensity domains.

These slopes were used to calculate the percentage increases above the first 180 s of exercise, as follows:

$$\rm{Y}_{(t)}=\left\{\left[{Y}_{180}+\left(Slope\times \left(t-180\right)\right)\right]\times 100\right\}/{Y}_{180}$$

where *Y*_(t)_ represents the value of a given variable at a given time coordinate above 180 s after exercise onset, *Y*_180_ is the mean value of the parameter between 170 and 180 s after exercise onset, Slope_Y_ is the slope of increase of a given parameter in the time window between 180 and 540 s and *t* is the time coordinate.

### Statistics

After assumptions verification (i.e. normality, homogeneity of variance), a two-way repeated-measures ANOVA was performed to assess differences over time in the 180–540-s time window and between different intensity domains (time segment × intensity domain) for ${\dot{V}O_2}{_m}$, [HHb], RMS, [La^−^], pH and HCO_3_^−^. Post hoc analysis was performed using the Holm-Sidak method. Moreover, partial eta squares (η_p_^2^) were calculated to quantify the effects sizes of different independent variables during the constant work rate trials following ANOVAs [25], and Cohen’s *d* was used to calculate effect sizes after multiple comparisons [11].

The linear relationships between the slope of the percentage change of ${\dot{V}O_2}{_m}$ and the percentage change of RMS and [HHb] were modelled, and Pearson’s product moment correlation coefficients were calculated. Finally, a multiple linear regression was run incorporating both RMS and [HHb] measured in each exercise intensity domain to test the combined muscle recruitment/metabolic instability contribution in predicting the slope of ${\dot{V}O_2}{_m}$ (i.e. ${\dot{V}O_2}{_s}{_c}$).

Based on an expected standard deviation of breath-by-breath ${\dot{V}O_2}$ measurements for steady-state exercise equal to 2.5% and a minimum detectable change in ${\dot{V}O_2}$ of 100–170 ml·min^−1^ at a ${\dot{V}O_2}$ of 2.1 to 3.5 L·min^−1^ [26], the minimum sample size to obtain a power of 0.8 was 6 individuals. Data are presented as means ± SD. All statistical analyses were performed using Sigmaplot version 12 and α was set in advance at the 0.05 level. Statistical significance was accepted when *p* < α.

## Results

**Fig. 1:**
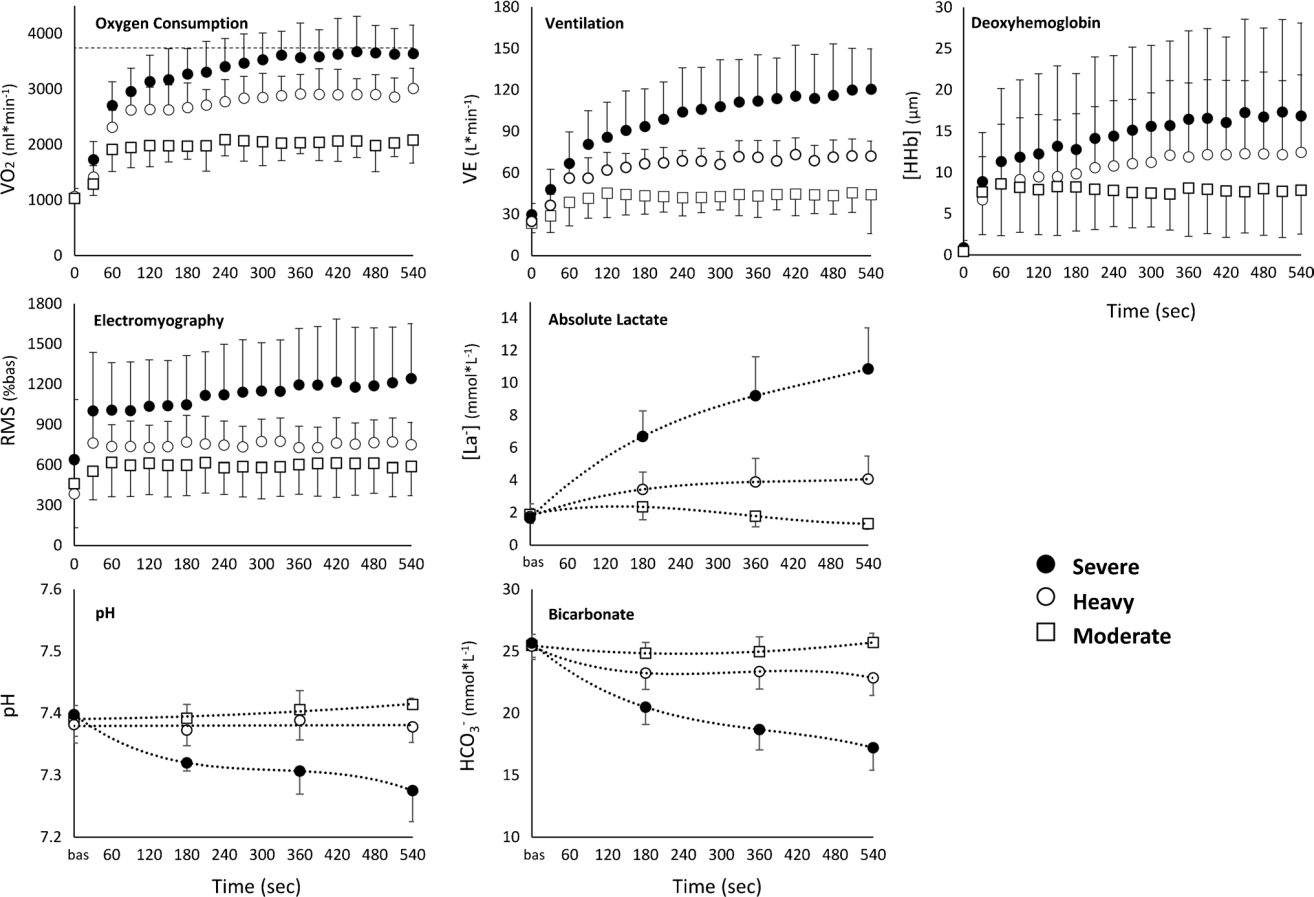
The physiological responses during cycling in different exercise domains are presented in 30 s means ± SD. Symbols represent: white square: moderate exercise domain, white circle: heavy exercise domain, black circle: severe exercise domain

GET and RCP were identified at a ${\dot{V}O_2}$ respectively of 2418 ± 385 and 3094 ± 377 ml*min^−1^, while the ${\dot{V}O_2}$ targets in the moderate, heavy and severe exercise domains were identified at 1935 ± 308 ml*min^−1^, 2743 ± 348 ml*min^−1^ and 3154 ± 408 ml*min^−1^.${\dot{V}O_2}$ values recorded in the last 30 s of exercise were 1973 ± 478 ml*min^−1^ for moderate, 3013 ± 365 ml*min^−1^ for heavy and 3640 ± 514 ml*min^−1^ for severe, highlighting a clear contribution of the ${\dot{V}O_2}$ slow component in the rise of ${\dot{V}O_2}$ over time both in the heavy and severe domains. The mean ${\dot{V}O_2}$ profile and the other physiological variables are represented in the three exercise intensity domains in Fig. 1.

**Fig. 2:**
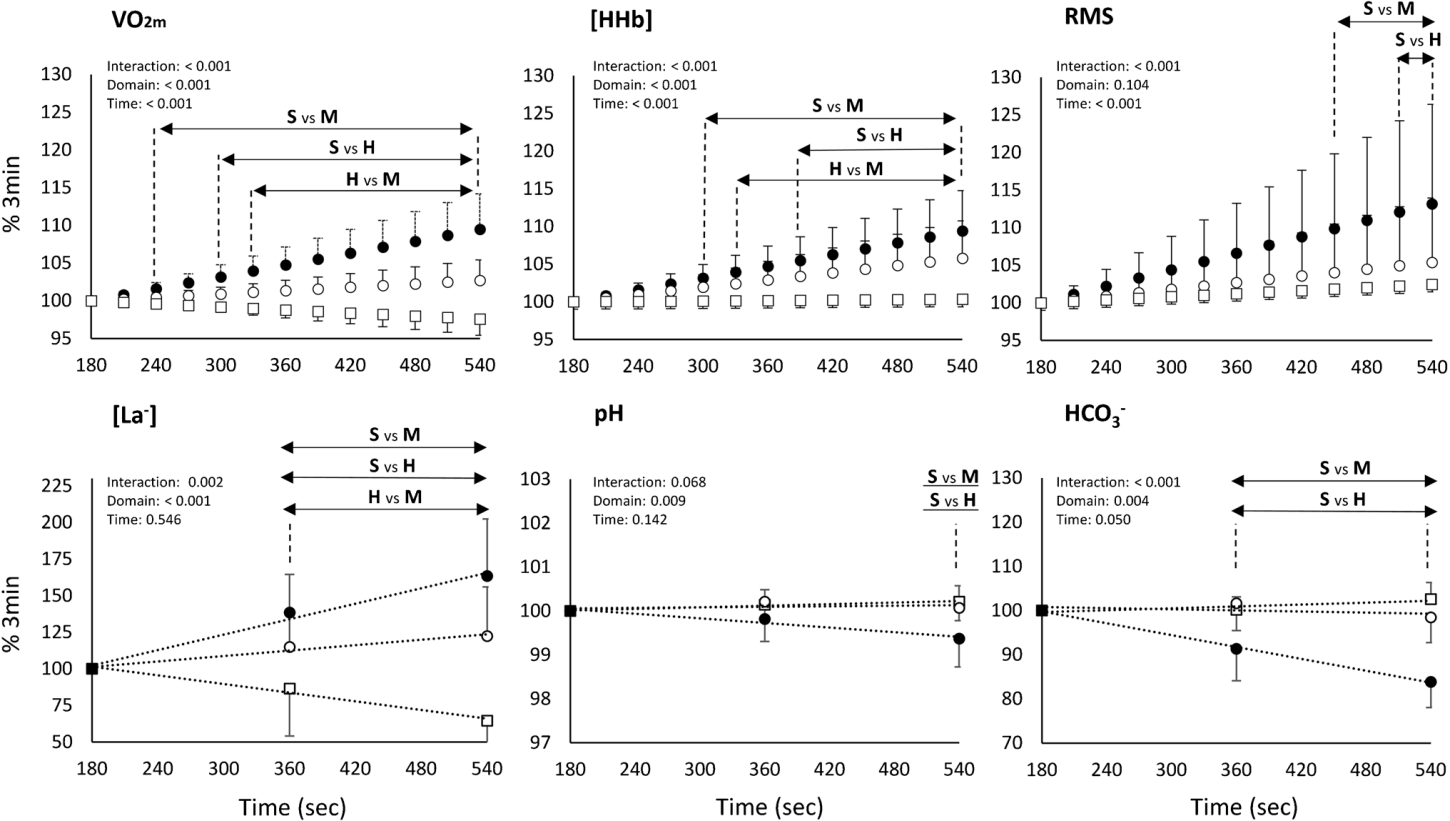
The dynamic changes of ${\dot{V}O_2}$, [HHb] and RMS after the third minute of exercise are represented in the top panels (as the % increase after the value reached the third minute of exercise). In the bottom panels, the changes of the haematic values of metabolic stability are displayed. Symbols represent: white square: moderate exercise domain, white circle: heavy exercise domain, black circle: severe exercise domain. Main effects resulting from ANOVA are represented in each panel, and the statistical differences resulting from the post hoc analysis are expressed by the letters (S vs M: severe vs moderate; S vs H: severe vs heavy; H vs M: heavy vs moderate)

In Fig. 2, the dynamic changes in these variables after 180 s of exercise are presented. ANOVAs revealed a significant “time” × “intensity domain” interaction for ${\dot{V}O_2}{_m}$ (*p* < 0.001, η_p_^2^: + 0.83), [HHb] (*p* < 0.001, η_p_^2^: + 0.67), RMS (*p* < 0.001, η_p_^2^: + 0.28), [La^−^] (*p* = 0.002, η_p_^2^: + 0.80) and HCO_3_^−^ (*p* < 0.001, η_p_^2^: + 0.69). On the contrary, no interaction (*p* = 0.068 η_p_^2^: + 0.42) and only a significant main effect of the intensity domain was detected for pH (*p* = 0.009, η_p_^2^: + 0.49). All the multiple comparisons between domains at different time points resulting from the post hoc analysis are presented in Fig. 2. In brief, ${\dot{V}O_2}{_m}$ and muscle [HHb] continued to increase over time after the first 180 s of exercise in severe (% difference between 540 and 180 s: ${\dot{V}O_2}{_m}$ + 9.4 ± 4.7%, *p* < 0.001, *d* + 2.0; [HHb] + 9.4 ± 5.4%, *p* < 0.001, *d* + 1.7) and in heavy (% difference between 540 and 180 s: ${\dot{V}O_2}{_m}$ + 2.7 ± 2.7%, *p* < 0.001, *d* + 1.0; [HHb] + 5.8 ± 4.9%, *p* < 0.001, *d* + 1.2) but not in moderate (${\dot{V}O_2}{_m}$ − 2.4 ± 2.1%, *p* = 0.001, *d* − 1.1; [HHb] + 0.3 ± 2.7%, *p* = 1.000, *d* + 0.1). Muscle activation increased over time only in severe (% difference between 540 and 180 s in RMS: severe + 13.1 ± 13.2%, *p* < 0.001, *d* + 1.0; heavy + 5.4 ± 8.5%, *p* = 0.110, *d* + 0.6; moderate + 2.5 ± 8.5%, *p* = 1.000, *d* + 0.3). The comparison between different domains revealed that ${\dot{V}O_2}{_m}$, [HHb] and RMS were different in severe from heavy and moderate, while heavy ${\dot{V}O_2}{_m}$ and [HHb] were the only signals to reach statistical significance versus moderate (Fig. 2). Regarding the haematic values, blood lactate concentration increased over time in severe (% difference between 540 and 180 s: + 63.4 ± 39.1%, *p* < 0.001, *d* + 1.62), remained stable in heavy (% difference between 540 and 180 s: + 22.3 ± 33.7%, *p* = 0.093, *d* + 0.66) and decreased in moderate (% difference between 540 and 180 s: − 35.5 ± 18.5%, *p* = 0.004, *d* − 1.91), while both pH and HCO_3_^−^ decreased over time in the severe domain only (% difference between 540 and 180 s in severe: pH − 0.6 ± 0.6%, *p* < 0.001, *d* − 0.98; HCO_3_^−^  − 16.1 ± 5.8%, *p* < 0.001, *d* − 2.78; heavy: pH + 0.1 ± 0.3%, *p* = 0.646, *d* + 0.24; HCO_3_^−^  − 1.6 ± 5.7%, *p* = 0.418, *d* − 0.27; moderate: pH + 0.2 ± 0.4%, *p* = 0.438, *d* + 0.59; HCO_3_^−^  + 2.5 ± 3.8%, *p* = 0.463, *d* + 0.68).

**Fig. 3:**
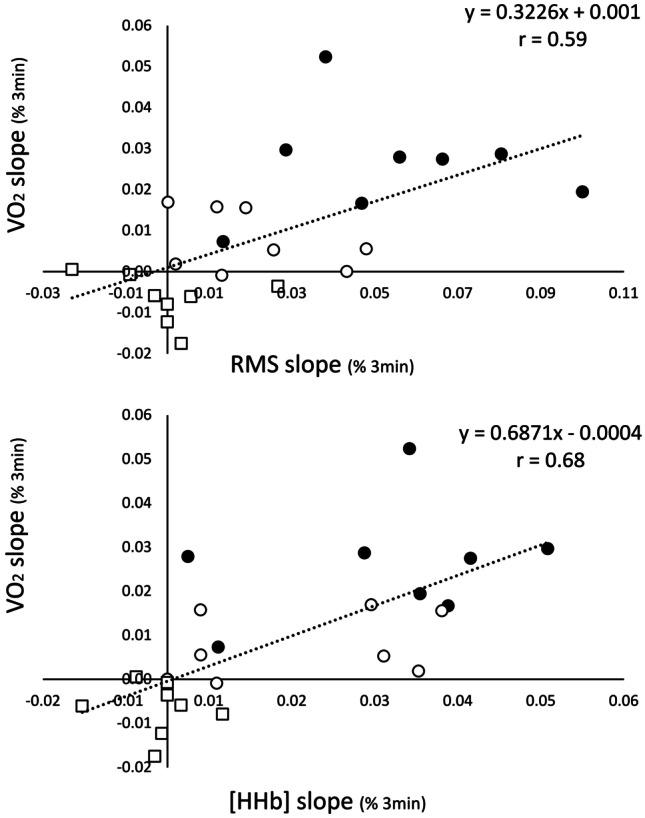
The correlations between the increases in RMS and [HHb] with ${\dot{V}O_2}{_m}$ are presented. Symbols represent: white square: moderate exercise domain, white circle: heavy exercise domain, black circle: severe exercise domain

Finally, the relationships of [HHb] and RMS with ${\dot{V}O_2}{_m}$ are presented in Fig. 3. All these variables showed a medium to high correlation with ${\dot{V}O_2}{_m}$ ([HHb]: *r* = 0.68, *p* < 0.001; RMS: *r* = 0.59, *p* = 0.002. Moreover, the multiple linear regressions showed that both [HHb] and RMS concurred to ${\dot{V}O_2}{_m}$ (*r* = 0.75, [HHb] *p* = 0.005, RMS *p* = 0.042), as described by the following equation:

$${\dot{V}}O_2=-0.00289+\left(0.520*\left[\rm{HHb}\right]\right)+(0.193*\rm{RMS})$$

where ${\dot{V}O_2}{_s}{_c}$ represents the slope of percentage increase in ${\dot{V}O_2}{_m}$ after the third minute of exercise; [HHb] represents the slope of % increase in deoxyhaemoglobin after the third minute of exercise and RMS represents the slope of percentage increase in root mean square after the third minute of exercise.

## Discussion

By implementing measures of peripheral metabolism and muscle activation, this study investigated the contribution of metabolic instability (i.e. [HHb], haematic markers) and increased muscle excitation (i.e. RMS) to the ${\dot{V}O_2}{_s}{_c}$ in the moderate, heavy and severe exercise intensity domains. In agreement with our hypothesis, our results showed that after the third min of exercise: (i) in the moderate domain, neither ${\dot{V}O_2}{_m}$ nor [HHb] or RMS increased over time; in heavy, an increase in ${\dot{V}O_2}{_s}{_c}$ occurred, in association with an increase in [HHb] but not in RMS; in severe, ${\dot{V}O_2}{_s}{_c}$ [HHb] and RMS all increased over time and the amplitude of these responses significantly differed from both moderate and heavy and (ii) the increases in [HHb] and RMS were significantly correlated with the increase in ${\dot{V}O_2}{_s}{_c}$. Overall, our findings are consistent with the hypothesis that the appearance of a loss of efficiency of locomotion over time, after the first 180 s, may be caused by a combination of metabolic instability within the working fibres and the increased muscle excitation.

In 2015, Grassi et al. provided a comprehensive, theoretical framework of the complex interplay of mechanisms underpinning the ${\dot{V}O_2}{_s}{_c}$ [17]; however, to this day, no study has provided experimental data on the changes and interactions of these mechanisms in different intensity domains. Therefore, this investigation contributed to fill this gap.

As expected, our data confirm no further increases in ${\dot{V}O_2}{_m}$ (− 2.4 ± 2.1%, *p* = 0.001), [HHb] (+ 0.3 ± 2.7%, *p* = 1.000) and RMS (+ 2.5 ± 8.5%, *p* = 1.000) after the third minute of exercise during moderate intensity transitions, indicating that a steady state was achieved in all these variables within 180 s.

In the heavy domain, a slow component of ${\dot{V}O_2}{_m}$ (+ 2.7 ± 2.7%, *p* < 0.001) ensued after the third minute and was accompanied by increased [HHb] (+ 5.8 ± 4.9%, *p* < 0.001) but only a small, non-significant increase in RMS (+ 5.4 ± 8.5%, *p* = 0.110). In this domain, the ${\dot{V}O_2}{_s}{_c}$ is typically described as a loss of efficiency over time; however, it was recently proposed that ${\dot{V}O_2}{_s}{_c}$_c _may, in fact, represent a delayed “metabolic shift” between anaerobic and aerobic sources for ATP resynthesis that occurs in coincidence with an increased cost of locomotion in the heavy domain of exercise (i.e. loss of efficiency vs moderate but not developing over time) [10]. Indeed, performing exercise in the heavy exercise domain entails the recruitment of bigger, intrinsically more glycolytic and less efficient motor units [32] which, in turn, may lead to slower ${\dot{V}O_2}$ kinetics [1] and larger ${\dot{V}O_2}$ gain (i.e. loss of efficiency of locomotion) [24] even without the recruitment of new muscle fibres over time. A possible physiological explanation for this phenomenon was proposed more than 30 years ago by di Prampero [14], and recently deepened by Korzeniewski and Zoladz [28]. The authors suggested that a larger contribution of glycolysis to ATP resynthesis (as during exercise above GET due to the recruitment of higher order muscle fibres) would slow the ${\dot{V}O_2}$ kinetics and justify the appearance of a delayed steady state or ${\dot{V}O_2}{_s}{_c}$. This possibility was also corroborated by a study showing an increase in the amplitude of the primary component of ${\dot{V}O_2}$ and, in turn, a reduction of the amplitude of the ${\dot{V}O_2}{_s}{_c}$, when heavy exercise was performed in a condition of glycogen depletion [5]. The possibility that the ${\dot{V}O_2}{_s}{_c}$ may in fact represent a delayed adjustment of ${\dot{V}O_2}$ seems therefore plausible, even if further studies will need to confirm this hypothesis. In support of this view, our data indicate increased levels of [HHb], compatible with increased metabolic instability within the working muscle/increased O_2_ cost, without augmented muscle activation (i.e. EMG) over time (Figs. 1 and 2). Moreover, [HHb] showed a slow component until the ~ 6th min of exercise, reaching a steady state thereafter (Fig. 1). It is reasonable to speculate that if a loss of efficiency over time would have occurred within the working motor units, its effects would have been protracted until the end of the exercise. In fact, the progressive accumulation of metabolites (P_i_, IMP, ADP, H^+^, K^+^) [17] would have probably led to a decreased efficiency/fatigue of the active fibres, to the recruitment of new fibres, and further moving away from homeostasis.

Finally, in the severe domain, ${\dot{V}O_2}{_m}$, [HHb] and RMS continued to increase over time even after the third minute of exercise (~ +9 to +13% between 180 and 540 s) (Fig. 2). This is an expected finding for exercise performed at an intensity in which a metabolic steady state is not achievable. Under these conditions, the aerobic energetic system alone is unable to sustain the required levels of ATP resynthesis and increased dependence on anaerobic metabolism is needed. This leads to the release of H^+^ and other metabolites [17] that impair muscle contraction and require the further contributions of larger, less efficient motor units to sustain exercise. As a result, an increasing level of metabolic perturbation occurs and the recruitment of higher order motor units is necessary to protract exercise [32].

A different development of metabolic instability over time in the three domains was also confirmed by the haematochemical markers of metabolic acidosis (Figs. 1 and 2). As expected, increasingly larger amounts of [La^−^] accumulated in the initial 3 min of exercise (early lactate) in the three domains, along with increasing reductions of blood pH and bicarbonate concentration. Thereafter, a steady state of these variables was maintained in the 3- to 9-min time window for both the moderate and heavy exercise domains. In the severe domain only, [La^−^] significantly increased (+ 63.4 ± 39.1%, *p* < 0.001), and both pH (− 0.6 ± 0.6%, *p* < 0.001) and HCO_3_^−^  − 16.1 ± 5.8%, *p* < 0.001) decreased over time, indicating a mismatch between blood lactate production and removal and an increasing metabolic acidosis. While changes in [La^−^] and acidosis between domains were recently shown by others [4, 35], this is the first study that concurrently performed non-invasive and time-resolved measures of ${\dot{V}O_2}{_m}$, local muscle metabolism, muscle activation and blood markers of metabolic instability.

The final aim of this investigation was to gain insight into the relative contribution of the two main putative physiological underpinnings of the ${\dot{V}O_2}{_s}{_c}$, metabolic instability and increased muscle recruitment. Our data indicate that [HHb] and RMS, both individually (Fig. 3) and combined in a multiple linear equation, had a significant impact on the prediction of the slope of the slow component after the initial 180 s. Moreover, the multiple linear equations indicated that the contribution to the ${\dot{V}O_2}{_s}{_c}$ was ~ 2.7 times larger for [HHb] than RMS, even if this quantitative indication should be taken carefully due to the different signal-to-noise ratio of the variables (higher for EMG) and to the presence of a slow component of RMS only in the severe domain (Figs. 1 and 2). To our knowledge, only two other studies provided data of simultaneous measurement of metabolic instability and muscle activation during constant work rate exercises in different intensity domains [4, 27]. In one of these studies, Keir et al. [27] found that the development of the ${\dot{V}O_2}{_s}{_c}$ in the severe domain was accompanied by peripheral muscle fatigue (i.e. reduced maximal isometric contraction), without increases in the EMG signal, concluding that metabolic instability was the only probable cause of the ${\dot{V}O_2}{_s}{_c}$. However, the lack of a slow component in the EMG response compared to our results may be explained by the different exercise intensities that were used in our studies. In Keir et al., the average ${\dot{V}O_2}$ reached at the 9th minute into exercise corresponded to ~  + 5% of the ${\dot{V}O_2}$ at RCP, i.e. in close proximity with the heavy-severe boundary [34], while in our study, it represented ~  + 17% of RCP. Pertinently with this speculation, recent findings showed that the muscle activation might change according to the relative intensity within the severe domain, with larger activation occurring at the higher intensities [21]. The relatively small dynamics of the ${\dot{V}O_2}{_s}{_c}$ reported in Keir’s study are also compatible with an intensity only slightly above the maximal lactate steady state [22] or the critical power [3, 6, 33]. The only other investigation that examined contemporarily neuromuscular and metabolic changes over prolonged exercise sessions reported a clear difference in the neuromuscular response during exercise in the severe vs heavy and moderate domains: fatigue/metabolic instability develops intensively in the severe domain (due to a large metabolic imbalance) and extensively in the heavy and moderate domains (due to substrates depletion and accumulation of fatigue-related metabolites) [4]. While their study is not easily comparable with ours due to difference in the design, Black’s data seem to indicate that a significant increase in metabolic imbalance and muscle activation was present in the initial 10 min of exercise only for the severe intensity domain.

This study has many possible implications for clinical practice and future research. In the former context, the interplay between ${\dot{V}O_2}{_s}{_c}$, muscle metabolic instability and increased muscle recruitment suggests that rehabilitation interventions aimed at improving both peripheral muscle metabolism (e.g. blood flow occlusion training, high-intensity training with considerable local stimulus) or muscle strength (e.g. resistance training) could mutually improve exercise tolerance. This could be important for patients who cannot achieve high whole-body exercise intensities due to central limitations (e.g. chronic heart failure, chronic obstructive pulmonary disease) but that could tolerate more peripherally focused exercises. In the latter context, identifying the contributors to the ${\dot{V}O_2}{_s}{_c}$ will help to understand the behaviour of ${\dot{V}O_2}$ kinetics in different exercise intensity domains that are used in a wide variety of studies. Future research will have to confirm these findings, possibly by implementing more invasive techniques. Moreover, the temporal sequence of muscle metabolic instability and fibre recruitment as well as which of these two mechanisms trigger the other remains to be determined.

In conclusion, this investigation tested experimentally the “instability-recruitment” theory proposed by Grassi et al. in 2015 [17] to explain the origins of the ${\dot{V}O_2}{_s}{_c}$. In the three exercise intensity domains, our study demonstrated a domain-dependent dynamic over time for ${\dot{V}O_2}$, [HHb] (taken as an index of metabolic instability), RMS (taken as an index of muscle excitation) and the whole-body haematochemical markers of metabolic instability. About 75% of the dynamics of the ${\dot{V}O_2}{_s}{_c}$ was explained by a combination of the dynamics of instability and recruitment, with metabolic instability having about threefold the relative weight compared to recruitment.
